# Active Aging From Theory to Practice: National Experiences of Policy Making in Europe and Canada

**DOI:** 10.1093/geroni/igab046.1660

**Published:** 2021-12-17

**Authors:** Francesco Barbabella

## Abstract

Born in Europe as a concept aiming to counteract new demographic and societal challenges, active aging has progressively become a key pillar of an extended welfare state for aging populations in many high-income countries. Needs, interests, and preferences of new aging cohorts are changing, becoming more diverse and requiring a better understanding and greater attention by policy makers, beyond mere social welfare programmes for those with social, economic or health needs. Active aging policies aim at improving individuals’ quality of life by optimizing opportunities for health, participation, and security (WHO 2002), hence unlocking the potential of older people as active citizens in the community and the society. Since the focus is on a multidimensional concept of quality of life, active aging works at the intersection of labour, social, educational, family, infrastructure, and many other policy areas. However, there may be gaps and discrepancies between the concept in itself and its application at the policy level. The purpose of this symposium is to present and discuss how different post-industrial societies are advancing and implementing active aging policies, in the context of overarching societal challenges and competing needs. In this respect, the symposium focuses on four countries representing different traditional welfare state models: Canada, Italy, Poland, and the United Kingdom. These four case studies bring analyses of active aging policies at national and/or regional level, providing a picture of how such policies have been designed, how they evolved and what they have achieved in recent years.

